# *TGFβ1*-transfected tendon stem cells promote tendon fibrosis

**DOI:** 10.1186/s13018-022-03241-y

**Published:** 2022-07-21

**Authors:** Hong-Bin Yu, Jing Xiong, Hui-Zhen Zhang, Qin Chen, Xu-Yong Xie

**Affiliations:** Department of Sports & Rehabilitation Medicine, The First People’s Hospital of Jiujiang City, No. 48 of Taling Street, Jiujiang District, Jiujiang, 332000 China

**Keywords:** Tendon stem cells, TGFβ1, Osteogenesis, Chondrogenesis, Adipogenesis

## Abstract

**Background:**

In aged people, tendon injuries frequently occur during sporting and daily activities. In clinical practice, typical physiotherapeutic, pharmacotherapeutic, and surgical techniques do not result in the full recovery of injured tendons, which may lead to chronic degenerative disease.

**Methods:**

We first isolated tendon stem cells (TSCs) from rats and transfected them with the *TGFβ1* gene, resulting in *TGFβ1*-TSCs. The proliferation of TSCs was detected using the Cell Counting Kit 8, and TSCs were identified by immunofluorescence analysis and differentiation capacity analysis. Aggrecan, COL2A1, alpha smooth muscle actin (α-SMA), and p-Smad2 expression levels were detected using western blotting and quantitative reverse transcription polymerase chain reaction. Additionally, a tendon injury model was generated to explore the effect of TGFβ1 on the repair of the tendon by TSCs.

**Results:**

Compared with fibrinogen treatment, TSC + fibrinogen or *TGFβ1*-TSC + fibrinogen treatment significantly promoted the fibrosis of injured tendons, as evidenced by histological analyses, with *TGFβ1*-TSC + fibrinogen having a greater effect than TSC + fibrinogen. In *TGFβ1*-TSCs, increased expression levels of aggrecan and COL2A1 indicated that TGFβ1 signaling induced chondrogenic differentiation. Meanwhile, the increased collagen and α-SMA protein levels indicated that TGFβ1 promoted fibrogenesis. Additionally, TGFβ1 stimulated the production of phosphorylated Smad2 in TSCs, which suggested that the chondrogenic and fibrogenic differentiation of TSCs, as well as tissue regeneration, may be associated with the TGFβ1/Smad2 pathway.

**Conclusion:**

*TGFβ1*-TSC therapy may be a candidate for effective tendon fibrosis.

## Introduction

Aged people frequently experience tendon injuries while performing sports or even daily activities. Tendon injuries account for > 40% of musculoskeletal diseases [1, 2]. In clinical practice, physiotherapy, pharmacotherapy, and surgery are the typical choices for tendon repair [3–5]. However, these treatments do not usually result in a fully recovered tendon, which may result in a chronic degenerative disease [6]. During recovery in adults, injured tendons do not rebuild normal tissue, but mainly produce scar tissue, which lacks tensile strength [7]. The failure to completely recover is due to the low regeneration capability of tenocytes, which is associated with the hypovascularity and low metabolism of tendon tissue [8, 9]. Therefore, the development of new therapies capable of stimulating the regeneration of injured tendons has been encouraged in recent decades.

Uncommitted stem cells are endowed with a high proliferation potential; they can differentiate into all types of cells under the appropriate conditions [10]. Stem cell-based therapies have attracted increasing interest for tendon healing [11]. In contrast to adult tendons, fetal tendon tissue is capable of regeneration after impairment, and injured tendons fully regain their former performance [6]. However, embryonic stem cell-based therapies have a great risk of tumor generation, in addition to the ethical issues regarding the harvesting of cells from embryos [12]. Induced pluripotent stem cells are an alternative for tendon healing without ethical issues, but tumorigenesis cannot be avoided [13]. In recent years, mesenchymal stem cells have been shown to be safer because their self-renewal and/or differentiation potential are relatively restricted compared with those of embryonic and induced pluripotent stem cells [11]. Mesenchymal stem cells were first identified in tendon tissue in 2007 and were named tendon stem cells (TSCs) [14]. Several studies have shown that TSC-based therapies substantially promote tendon healing and tissue regeneration [15–17]. Stem cell-based treatment is the most promising therapeutic strategy for tendon healing [18, 19].

Previous studies have shown that tendon development and healing are highly associated with growth factors, such as fibroblast growth factor, epidermal growth factor, bone morphogenetic protein, and transforming growth factor (TGF) [11, 20]. Of these, TGFβ1 is one of the most attractive bioactive factors because it plays multiple roles in tendon healing [20]. Specifically, TGFβ1 is essential for tendon formation as it induces the expression of tendon-specific proteins and stimulates chondrogenic differentiation [21, 22]. TGFβ1 is also involved in the production of mesenchymal stem cells, it stimulates the production of collagen types I and III, and it participates in cell migration and mitogenesis [23, 24]. The application of stem cell-based therapies, in combination with growth factor supplementation, is being increasingly applied to tendon healing [11]. Nonetheless, information on the effects of TSC-based therapy in combination with TGFβ1 supplementation on tendon healing remains limited. Therefore, we isolated TSCs and overexpressed TGFβ1 in these cells to explore the effect of TGFβ1 on TSC-induced fibrogenesis and the expression of related molecules.

## Methods

### TSC isolation and culture

All animal experiments were approved by the animal research ethics committee of the First People’s Hospital of Jiujiang City. TSCs were collected from male Sprague-Dawley rats weighing approximately 50 g. Prior to TSC collection, the rats were anesthetized by intraperitoneal injection of 0.03% pentobarbital sodium (30 mg/kg), and the tendons were separated from the paratendon, fat, and muscle tissues. The tendon samples were sectioned into 1 mm slices and digested in a 3 mg/mL collagenase solution (Sigma-Aldrich, St. Louis, MO, USA). The digested solution was filtered through a 70 μm cell strainer and centrifuged at 300 × *g* for 5 min to collect the tendon cells. The cells were cultured in Dulbecco’s modified Eagle’s medium (Gibco, Invitrogen, Grand Island, NY, USA) supplemented with 10% fetal bovine serum (Beyotime Biotechnology, Shanghai, China) and 1% penicillin-streptomycin antibiotic (Beyotime Biotechnology), and were sub-cultured after reaching 80% confluence.

### Cell proliferation assay

Cells at passage three were seeded into 96-well plates (10^4^ cells per well) and incubated with Cell Counting Kit-8 solution (Beyotime Biotechnology) according to the manufacturer’s instructions. After 4 h, cell proliferation was assessed by measuring absorbance at 450 nm (SpectraMax; Molecular Devices, San Francisco, CA, USA).

### Immunofluorescence analysis

Cells at passage three were incubated with fluorescein isothiocyanate-conjugated antibodies against surface markers, including anti-CD34 (Abcam, Cambridge, UK), anti-CD73 (BioLegend, San Diego, CA, USA), and anti-CD90 (BioLegend). Fluorescence was detected using a flow cytometer (NovoCyte 1300; ACEA, San Diego, CA, USA) to identify TSCs.

To detect collagen type I, TSCs were fixed with 4% paraformaldehyde (catalog number: P0099, Beyotime Biotechnology) and blocked with 20% heat-inactivated horse serum (Gibco) supplemented with 0.1% Triton-X 100 (Sigma-Aldrich). The cells were incubated with an anti-collagen type I antibody (catalog number: 14695-1-AP; Proteintech, Wuhan, China), followed by fluorescein-conjugated goat anti-rabbit IgG (catalog number: SA00003-11, Proteintech). The cells were then stained with 4′,6-diamidino-2-phenylindole (catalog number: P0131, Beyotime Biotechnology). Fluorescence was visualized under a fluorescence microscope (Leica, Wetzlar, Germany).

### Differentiation capacity analysis

To evaluate multipotency, cells at passage three were sub-cultured into adipogenic, chondrogenic, or osteogenic media (Thermo Scientific, Pittsburgh, PA, USA). After 21 days of induction, the cells were fixed with 4% paraformaldehyde, washed with deionized water, and stained with alizarin red (osteogenic potential), alcian blue (chondrogenic potential), or oil red O (adipogenic potential). The stained cells were observed under a light microscope (Olympus, Tokyo, Japan).

### Generation of a TSC line stably overexpressing TGFβ1

*TGFβ1* cDNA was cloned and inserted into the pCDH-MCS-T2A-puro lentiviral vector (XIAMEN Anti-hela Biological Technology Trade Co. Ltd., Xiamen, China). The following primers were used: *TGFβ1* forward primer, 5′-TAGAGCTAGCGAATTCGCCACCATGATGCCGCCCTCGGGGCTGCG-3′ and TGFβ1 reverse primer, 5′-CAGCGGCCGCGGATCCGCTGCACTTGCAGGAGCGCAC-3′. The *TGFβ1* expression plasmid (pCDH-*TGFβ1*) or the pCDH-MCS-T2A-puro lentiviral vector (negative control) was transfected into HEK293T cells together with psPAX2 (a packaging plasmid) and pMD2.G (an envelope plasmid) using Lipofectamine 2000 (Life Technologies, Carlsbad, CA, USA). Cell supernatants containing lentivirus were harvested at 48 h post-transfection. Subsequently, the lentivirus was purified and concentrated and the median tissue culture infectious dose (TCID_50_) of the lentivirus was determined, as previously described [25]. For stable transfection, TSCs were incubated with 10^8^ TCID_50_/mL lentiviral particles to which 8 mg/mL polybrene had been added. At 3 days post-infection, the cells were treated with 1 mg/mL puromycin for 2 weeks. TGFβ1 overexpression was assessed by quantitative reverse transcription polymerase chain reaction (qRT-PCR), and the resulting cell line was named *TGFβ1*-TSCs.

### Total RNA extraction and first-strand cDNA synthesis

Total RNA was isolated from TSCs using TRIzol reagent (Takara, Dalian, China), following the manufacturer’s instructions. RNA quality and quantity were determined using 1% agarose gel electrophoresis and spectrometry (Nanodrop 2000, Thermo Scientific), respectively. RNA samples were treated with DNase I and reverse transcribed with random primers, a dNTP mix, and M-MLV reverse transcriptase (Takara) to synthesize first-strand cDNA.

### Quantitative PCR

qPCR was used to determine the expression levels of aggrecan, *COL2A1*, *TGFβ1*, and *RNA18S5N* (internal control). Reactions were performed on a QuantStudio 7 Flex (Thermo Scientific) using a SYBR Green PCR premix (Takara). The qPCR program was set as follows: a preheating step at 95 °C for 60 s; followed by 40 cycles of heating (95 °C for 30 s), annealing (58 °C for 35 s), and extension (72 °C for 60 s); and a final extension (72 °C for 10 min). Relative mRNA expression levels were determined using the 2^−ΔΔCT^ method. The following primers were used for qPCR: *TGFβ1* forward primer, 5′- CCGCAACAACGCAATCTA-3′; *TGFβ1* reverse primer, 5′- TGCTTCCCGAATGTCTGA-3′; *COL2A1* forward primer, 5′-GGAAGAGCGGAGACTACT-3′; *COL2A1* reverse primer, 5′- TCCATGTTGCAGAAGACTT-3′; aggrecan forward primer, 5′- CTTCTGCCTCTGGAATAG-3′; aggrecan reverse primer, 5′-CACTGACATCCTCTACTC-3′; *RNA18S5N* forward primer, 5′- AGGCGCGCAAATTACCCAATCC-3′; and *RNA18S5N* reverse primer, 5′-GCCCTCCAATTGTTCCTCGTTAAG-3′.

### Western blotting

TSCs were lysed with radioimmunoprecipitation assay buffer containing protease and phosphatase inhibitors (Beyotime Biotechnology). Isolated proteins were separated by 12% sodium dodecyl sulfate-polyacrylamide gel electrophoresis and transferred to polyvinylidene difluoride membranes (Bio-Rad, Hercules, CA, USA). After blocking with 5% skimmed milk, the membranes were incubated with antibodies against phosphorylated-smad2 (p-smad2) (catalog number: ab280888, Abcam), collagen type II (catalog number: ab34712, Abcam), aggrecan (catalog number: ab3778, Abcam), GAPDH (catalog number: 60004-1-Ig, Proteintech), or alpha smooth muscle actin (α-SMA) (catalog number: ab5694, Abcam), followed by incubation with horseradish peroxidase (HRP)-conjugated goat anti-mouse IgG (catalog number: SA00001-1, Proteintech) or goat anti-rabbit IgG (catalog number: SA00001-2, Proteintech). The protein bands were visualized using an HRP chemiluminescence kit (Immun-Star^TM^, Bio-Rad) on an ImageQuant LAS 4000 system (GE Healthcare, Hino, Japan).

### Surgical procedure and treatment

Adult male Sprague-Dawley rats weighing approximately 200 g were anesthetized by intraperitoneal injection of 0.03% pentobarbital sodium (30 mg/kg), and one-third of the patellar tendon was removed to mimic tendon injury following a well-established protocol [16, 26]. Fibrinogen, TSCs and fibrinogen (TSCs + fibrinogen), or *TGFβ1*-TSCs and fibrinogen (TGFβ1-TSCs + fibrinogen), in combination with thrombin, were injected into the defect area. Four weeks after surgery, the rats were killed for histological examination.

### Histological examination

Tendon samples were fixed with 4% paraformaldehyde for 24–48 h. The fixed samples were dehydrated, embedded in paraffin, and sectioned. The sections were stained with hematoxylin and eosin (H&E) and observed under a light microscope (BX51, Olympus).

### Statistical analysis

Statistical analyses were performed using Graph Pad Prism 5.0 (San Diego, CA, USA). An independent Student’s t-test was used for comparisons between groups, and data normality was verified using the Shapiro-Wilk test. Statistical significance was set a *p *< 0.05.

## Results

### TSC identification

**Fig. 1:**
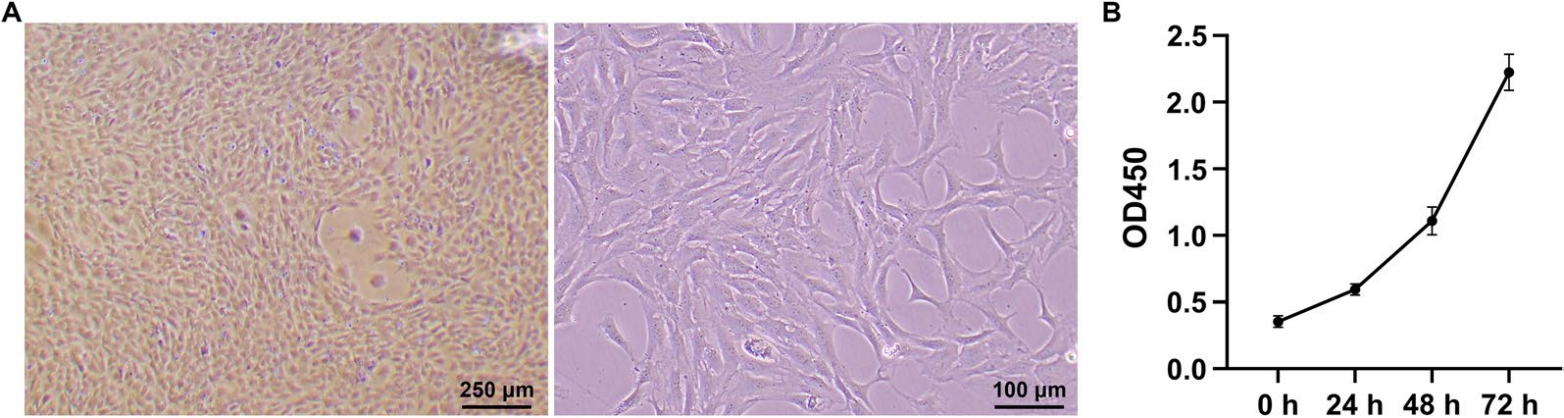
Morphology and proliferation of tendon stem cells. **A** Morphological observation of isolated cells. **B** Cell proliferation was assessed using Cell Counting Kit-8.

**Fig. 2:**
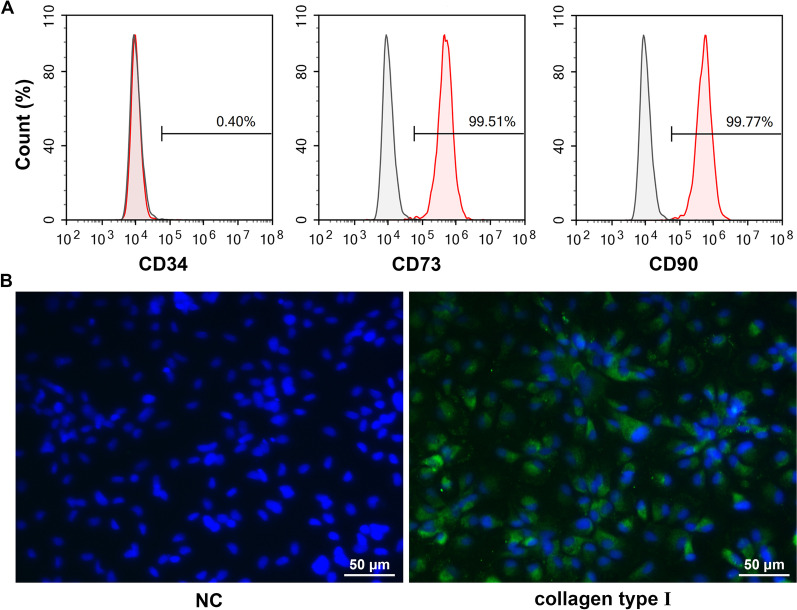
Expression of surface markers in tendon stem cells. **A** The expression of surface markers, including CD34, CD73, and CD90, was detected by flow cytometry. **B** The expression of collagen type I was detected by immunofluorescence. NC: cells did not incubate primary antibodies.

**Fig. 3:**
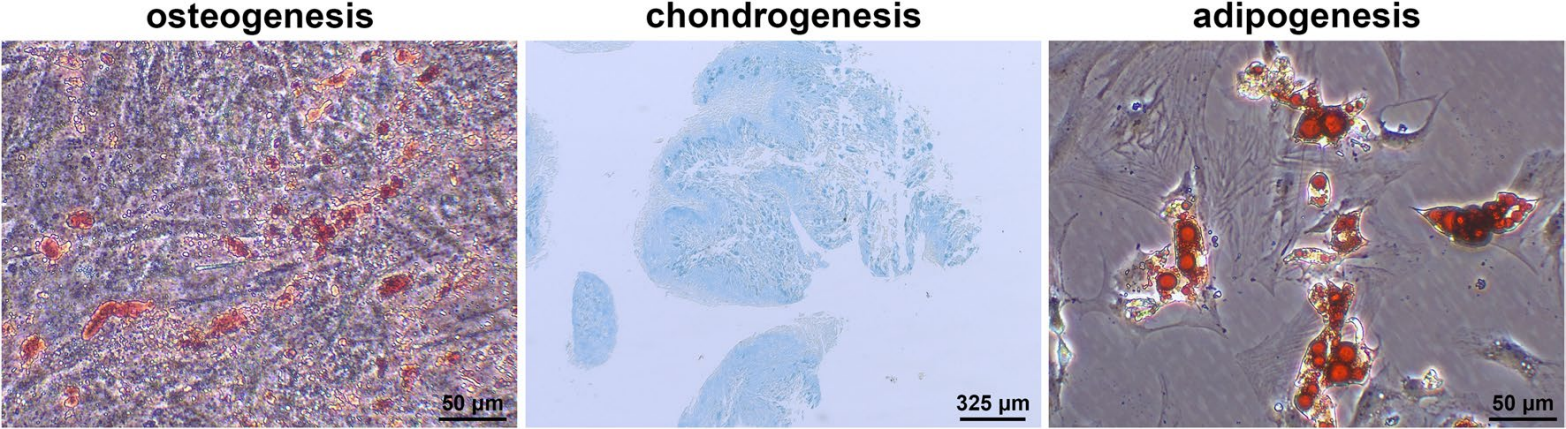
Evaluation of tendon stem cell differentiation capacity. Alizarin red, alcian blue, and oil red O staining showing osteogenic, chondrogenic, and adipogenic potential, respectively.

Cells obtained from rat tendons were large, flat, and fibroblastic (Fig. 1A) and cell proliferation increased from 0 to 72 h of culture (Fig. 1B). Subsequently, we detected the surface markers on the TSCs. The isolated cells were negative for the hematopoietic stem cell-like marker, CD34 (0.40%). However, they were positive for the mesenchymal stem cell-like markers, CD73 (99.51%) and CD90 (99.77%, Fig. 2A). In addition, the isolated cells were positive for collagen type I expression, indicating that they were derived from tendons (Fig. 2B). To confirm the identity of the isolated cells as TSCs, we performed differentiation experiments. Osteogenic differentiation was confirmed by the presence of calcium deposits and alkaline phosphatase activity using alizarin red staining. Chondrogenic and adipogenic differentiation were determined by the presence of sulfated glycosaminoglycan using alcian blue staining and the presence of oil droplets using oil red O staining (Fig. 3). These results showed that the isolated cells could differentiate into osteoblasts, chondrocytes, and adipocytes, indicating that we had successfully isolated TSCs.

### TGFβ1 overexpression in TSCs increases the expression of chondrogenic and fibrogenic markers

**Fig. 4:**
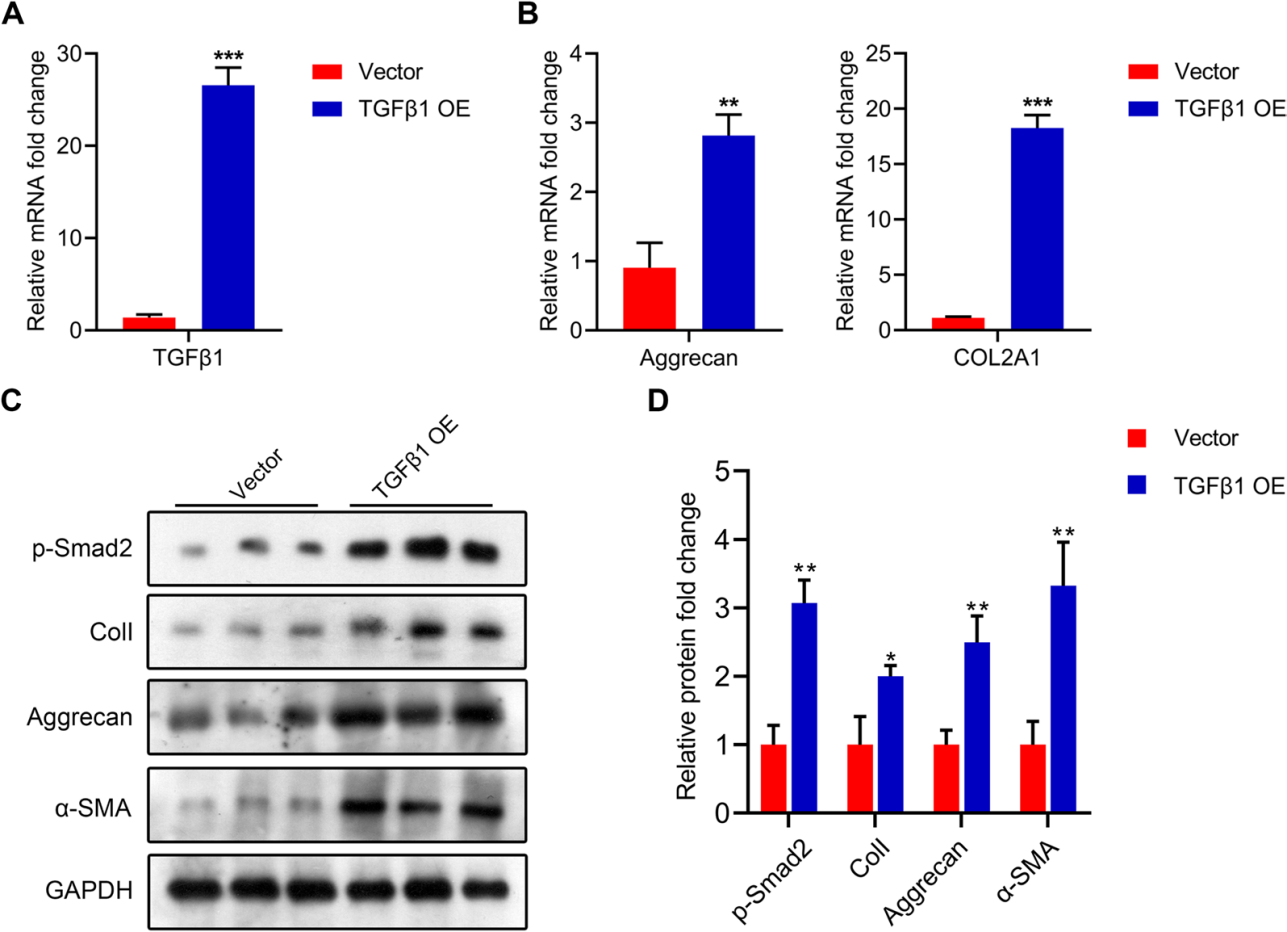
TGFβ1 overexpression in tendon stem cells increases the expression levels of chondrogenic and fibrogenic markers. **A** The mRNA level of *TGFβ1* in *TGFβ1*-TSCs. **B** The mRNA levels of aggrecan and *COL2A1* in *TGFβ1*-TSCs. **C**, **D** The protein levels of phosphorylated (p)-Smad2, collagen type II (Coll), aggrecan, and α-SMA in *TGFβ1*-TSCs. TSC: tendon stem cell; OE: overexpression. *p < 0.05, **p < 0.01, ***p < 0.001.

After the transfection of pCDH-*TGFβ1*, the expression of *TGFβ1* was successfully detected in TSCs (Fig. 4A). The relative expression levels of the chondrogenic markers, aggrecan and *COL2A1*, in TSCs significantly increased upon TGFβ1 overexpression (Fig 4B). Furthermore, the protein levels of the fibrogenic and chondrogenic markers, collagen type II, α-SMA, p-smad2, and aggrecan significantly increased in TSCs upon TGFβ1 overexpression (Fig. 4C).

### TGFβ1 overexpression enhances TSC-mediated tendon fibrosis

**Fig. 5:**
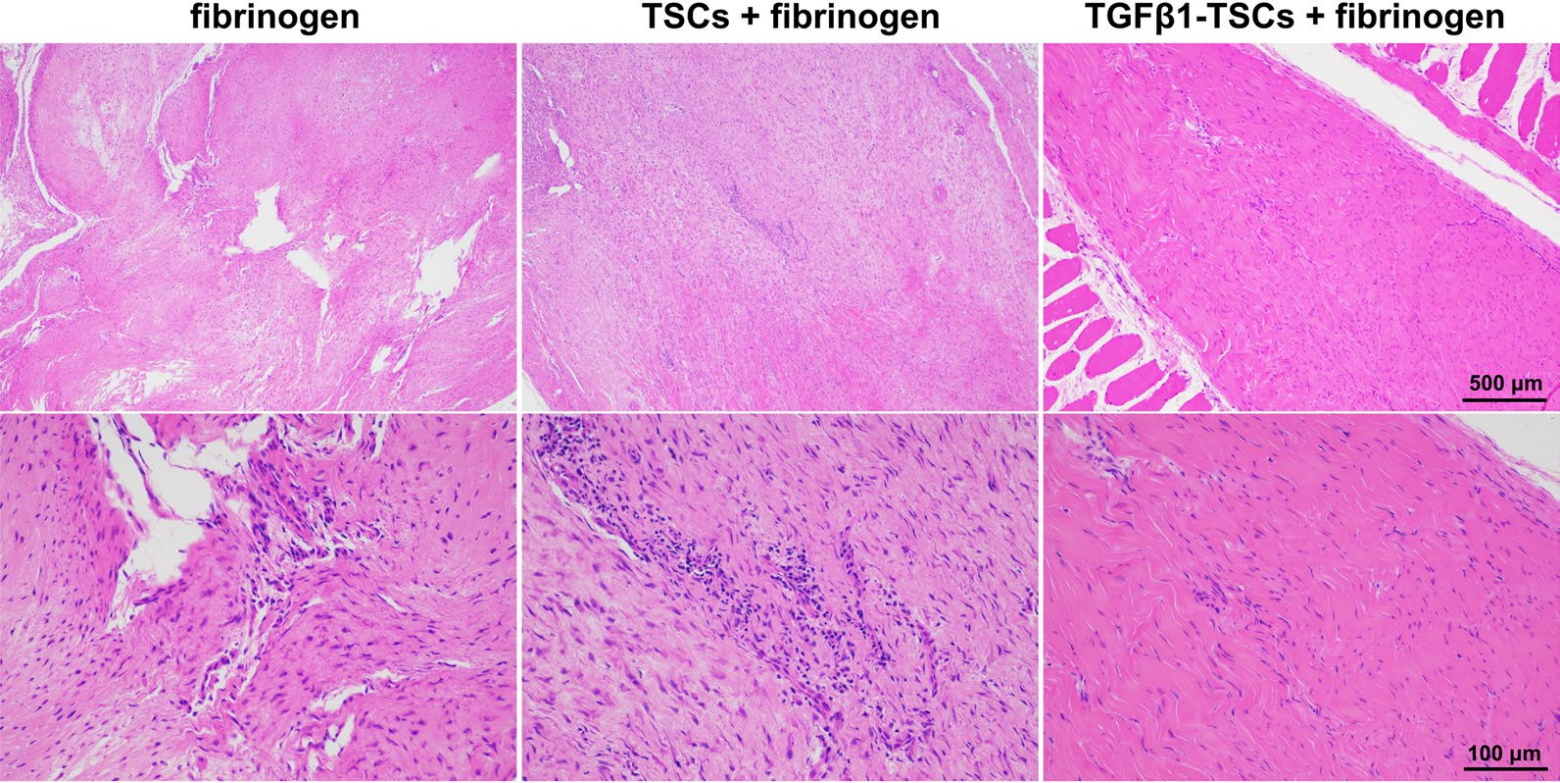
*TGFβ1*-transfected TSCs promote tendon healing. Hematoxylin and eosin staining of tendon sections treated with fibrinogen, TSCs + fibrinogen, or *TGFβ1*-TSCs + fibrinogen.

H&E staining showed that rupture and macrophage infiltration were still observed in tendons treated with fibrinogen (Fig. 5). Macrophage infiltration was observed in TSCs + fibrinogen-treated tendons. In contrast, the tendons treated with *TGFβ1*-TSCs + fibrinogen were continuous, without macrophage infiltration. These findings suggest that TGFβ1 enhanced the tendon-fibrosis ability of TSCs.

## Discussion

Tendon recovery was promoted by treatment with TSCs + fibrinogen or *TGFβ1*-TSCs + fibrinogen, both of which had a greater effect than treatment with fibrinogen alone. The observed improvement in tendon recovery confirmed that TSC-based therapies substantially promote tissue regeneration after injury, which is in agreement with previous findings [15–17]. Moreover, fibrosis was significantly higher in the *TGFβ1*-TSCs + fibrinogen group than in the TSCs + fibrinogen group. Our findings are consistent with those of a previous study of TSC-based therapies supplemented with exosomes or extracellular matrix containing TGFβ1 [27, 28]. These findings suggest that *TGFβ1*-TSC therapy may be a candidate for effective tendon healing. Furthermore, during the process of tissue injury healing, an appropriate amount of TGFβ1 promotes healing, whereas excessive TGFβ1 leads to scar hyperplasia and adverse effects on healing [29]. The amount of TGFβ1 produced by *TGFβ1*-TSCs was not determined, which is a limitation of this study. TGF-β1 signaling is regulated at multiple levels to avoid detrimental outcomes for cells [29]; thus, it is understandable that *TGFβ1*-TSCs promoted tendon repair, and this result indicates that the amount of TGFβ1 produced by *TGFβ1*-TSCs was appropriate.

Compared with TSCs, *TGFβ1*-TSCs showed higher expression levels of aggrecan and COL2A1. The increased expression levels of these two chondrogenic markers indicated that TGFβ1 signaling promoted the differentiation of TSCs into the chondrogenic lineage [30]. Similarly, the protein levels of collagen type II and α-SMA were also increased in TSCs upon TGFβ1 overexpression, which indicated that TGFβ1 promoted fibrogenesis in TSCs [31]. The increased protein levels of collagen type II and α-SMA suggested the increased production of extracellular matrix components that are dedicated to tendon structure formation [32]. Additionally, p-Smad2 levels were increased by TGFβ1 in TSCs, indicating that chondrogenesis and fibrogenesis in TSCs were induced via the TGFβ1/Smad2 pathway [33, 34]. These findings suggest that the TGFβ1/Smad2 pathway induced chondrogenic and fibrogenic differentiation of TSCs and promoted tissue regeneration and tendon healing.

## Conclusions

This study describes the molecular mechanism whereby TGFβ1 enhances the reparative effect of TSCs on tendons *in vivo*. Our results showed that TGFβ1 increased the levels of aggrecan, COL2A1, α-SMA, and p-Smad2 in TSCs. Furthermore, we demonstrated that *TGFβ1*-TSCs have a positive effect on the fibrosis of damaged tendons.

## Data Availability

The data that support the findings of this study are available on request from the corresponding author.
